# Single-Cell RNA-Seq Analysis Reveals Microenvironmental Infiltration of Plasma Cells and Hepatocytic Prognostic Markers in HCC With Cirrhosis

**DOI:** 10.3389/fonc.2020.596318

**Published:** 2020-11-02

**Authors:** Siwen Zhang, Zhenhao Liu, Dan Wu, Lanming Chen, Lu Xie

**Affiliations:** ^1^ Key Laboratory of Quality and Safety Risk Assessment for Aquatic Products on Storage and Preservation (Shanghai), China Ministry of Agriculture, College of Food Science and Technology, Shanghai Ocean University, Shanghai, China; ^2^ College of Food Science and Technology, Shanghai Ocean University, Shanghai, China; ^3^ Shanghai Center for Bioinformation Technology, Shanghai Academy of Science and Technology, Shanghai, China; ^4^ Key Laboratory of Carcinogenesis and Cancer Invasion, Ministry of Education, Key Laboratory of Carcinogenesis, National Health and Family Planning Commission, Xiangya Hospital, Central South University, Changsha, China; ^5^ Center for Biomedical Informatics, Shanghai Children’s Hospital, Shanghai Jiao Tong University, Shanghai, China

**Keywords:** single-cell RNA-seq, hepatocellular carcinoma, liver cirrhosis, plasma cells, humoral immunity

## Abstract

The occurrence of hepatocellular carcinoma (HCC) related to liver cirrhosis is mostly accompanied by extensive immune infiltration. To reveal the infiltration immune cells landscape, single-cell RNA sequencing data from the healthy donor (HD), patients with liver cirrhosis (LC) and HCC were collected for analysis. By drawing a cell map and calculating the proportion of each cell type, total B cells were identified with a significant higher proportion in HCC (24.26%) than in LC (5.41%) and HD (5.82%), in which plasma cells account for 97.1% in HCC. While in HCC, TCGA datasets were taken for further investigation, and it was found that patients with lower proportion of plasma cells showed better prognosis. The pseudotime cell trajectory analysis of B cell population found that humoral immunity continuously changes during HD, LC and HCC, and humoral immune-related genes are highly expressed in the HCC stage. This suggests humoral immunity may play a key role in the development of LC-associated HCC. At the same time, single cell data of hepatocytes identified differentially expressed genes in HD/LC and LC/HCC groups, and a prognostic model constructed with six of the differential genes (FTCD, MARCKSL1, CXCL3, RGS5, KNG1, and S100A16) could classify HCC patients to two distinct risk groups (median survival time 2.46 years vs. 6.73 years, p < 0.001). Our study demonstrated the power of single-cell data analysis in dissecting tissues into infiltration and main body cells, it revealed the pivotal roles of humoral immunity infiltration in the landscape of HCC associated with cirrhosis, and the key tumor prognostic genes in hepatocytes themselves. These brought novel insights into studying microenvironment and tumor cells parallelly in cancer research. The interaction of both, rather than factors from one side may have caused tumorigenesis and progression.

## Introduction

Liver cancer is the third leading cause of cancer-related mortality in the world (1), and hepatocellular carcinoma (HCC) accounts for approximately 90% of the incidence of all liver cancers (2, 3). The niche of liver tissue includes various cell lines such as hepatocytes, endothelial cells, fibroblasts, epithelial cells, and immune cells. In this unique ecosystem, tumor formation requires coordinated functions and crosstalk between specific cell types. Compared with other types of cancer, the HCC-compliant tumor microenvironment has a strong dependence on the number and state of immune cells (4), resulting in a lack of clinical success in the treatment of HCC. Recent advances in this area have demonstrated that many factors are related to the clinical response to checkpoint therapy (5). Unfortunately, these results are implicit and have not been further developed as clinical biomarkers (6). A detailed understanding of the various immune cells in the tumor microenvironment (TME) is essential for the development of effective immunotherapy for HCC and the identification of new biomarkers.

The immune microenvironment of HCC is a mixture of heterogeneous immune components (7, 8), immune cells migrate from the hematopoietic organ to the liver, establish an active immune niche that interacts with stromal cells, and affect differentiation, tumorigenesis and development. Therefore, it is important to explore the composition and state of immune cells during tumorigenesis. T cells and B cells are the most abundant and most characteristic cell groups in solid tumor TME (6). B cells are always important cellular components in the tumor microenvironment, and play a role through antibody production, antigen presentation and immune regulation. Some studies have shown that in human solid tumors such as breast cancer, oral cancer, non-small cell lung cancer (NSCLC), ovarian cancer and melanoma, the B cells that infiltrate the tumors are associated with a good prognosis (5). However, as a key factor in humoral immune response, the clinical relevance and prognostic significance of B cells and their subsets in HCC microenvironment are unclear.

HCC is highly heterogeneous, causing current curative effect restricted (9). The bulk omics analysis technique is to sequence the tissue blocks, and for the cell types with low richness, the complexity of cells and intracellular changes will be obscured (4). Single-cell isolates from individual tumor tissues contain carcinoma and non-carcinoma microenvironment cells (10). Each cell population has a unique pattern of gene expression, which cannot be resolved in the total mixed population (10). Single cell RNA-seq, which can resolve expression pattern of the unique cell population, makes it possible to study the relationship between unique subtypes and diseases.

Single cell sequencing technology (such as single cell RNA-seq, scRNA) has been applied in liver cancer or liver cirrhosis studies. Emerging data indicate that the mortality rate of HCC associated with cirrhosis is rising in some developed countries, whereas mortality from HCC with no cirrhosis complication is decreasing or is stable (11). Cohort studies indicate that HCC is the major cause of liver-related death in patients with concurrent cirrhosis (11). Cirrhosis from any cause predisposes to hepatocellular carcinoma (HCC) and hence can be considered a premalignant condition. Indeed, the majority of patients worldwide with HCC have underlying cirrhosis (12). On a global basis, some common risk factors lead to inflammatory fibrosis or cirrhosis, accompanied by extensive immune infiltration, which is considered to be a key factor in the development and progression of HCC (7).

In this study, the map of liver tissue cells and a small number of peripheral blood cells were drawn from three sources: healthy donors (HD), patients with liver cirrhosis (LC), and patients with hepatocellular carcinoma (HCC). Our results illustrated the role of B cells in the microenvironment of HCC, and simulated the development trajectory of B cells in three sources, providing a new insight into the impact of B cells on hepatocellular carcinoma. We also analyzed the impact of key genes of hepatocytes in this process that lead to the development of liver cirrhosis into liver cancer by prognostic analysis of patients. Our study may bring novel insights for the surveillance and treatment of cirrhosis-related HCC.

## Materials and Methods

### Data Collection and Preprocessing

To describe the landscape of the composition and functional states of hepatocellular carcinoma (HCC) during tumor progression, we collected single cell transcriptome datasets from liver tissues of healthy donors, patients with cirrhosis and HCC. The liver tissue cells of healthy donors and patients with cirrhosis come from GSE136103 (13), and the liver tissue cells of patients with HCC come from GSE125449 (14) (Downloaded from GEO database). By sampling and down-sampling to balance datasets, 3,913 hepatocellular carcinoma cells of tumor tissue, 10,000 cells of liver tissues from healthy donors, and 10,000 cells from patients with liver cirrhosis were included and incorporated. Alignment, tagging, gene, and transcript counting analysis of the two datasets were performed using the Cell Ranger single-cell software suite from 10X Genomics (GRCh38). Seurat (version 3.1.1) (15) was performed to pre-process the collected data, genes expressed in fewer than three cells in a sample were excluded, as well as cells that expressed fewer than 200 genes or cells with mitochondrial gene content >10% of the total unique molecular identifier (UMI) count.

### Data Integration, Clustering, and Cell Type Identification

To integrate cells into a shared space from different datasets for unsupervised clustering, *FindIntegrationAnchors* function and *IntegrateData* function were used to identify anchors and run integration step and eliminate batch effect. Then unsupervised clustering and differential gene expression analyses were performed. Based on the shared nearest neighbor module optimization algorithm, the first 20 PCs (principal components) were applied for UMAP (Uniform Manifold Approximation and Projection) analysis according to the eigenvalues (data not shown). Further, cells were clustered by the *FindClusters* function with the resolution parameter of 0.2. Next, through the function *FindAllMarkers*, groups of over expressed genes were identified to find subclusters. All UMAP visualizations, violin plots, and feature plots in the paper were produced using Seurat functions in conjunction with the ggplot2 (version 3.2.1), and pheatmap (version 1.0.12) R packages. Finally, we used the R package SingleR (version 1.0.5) (16) and scHCL (version 0.1.1) (17) to annotate cell types, and the annotation results were kept as a reference.

### Trajectory Analysis

In order to study the development trajectory of hepatocytes in the process of tumor development and progression, monocle (version 2.14.0, for pseudotime analysis) (18) was used to analyze the gene expression matrix with Seurat annotation. We screened the differentially expressed genes between HD and LC and between LC and HCC in B cells, arranged the cells in pseudo-time along the trajectory, drew heatmaps according to the genes of each branch of the trajectory, and divided them into five groups according to gene expression patterns.

### GO and KEGG Pathway Functional Enrichment Analysis

Gene Ontology (GO) annotation and Kyoto Encyclopedia of Genes and Genomes (KEGG) pathway enrichment analyses were performed by hypergeometric distribution using R package clusterProfiler (19) (version 3.14.3). The adjusted p-value were calculated using Benjamini and Hochberg method (20). P.adjust value < 0.05 was considered significant. Graphic visualization was implemented with function *dotplot* in clusterProfiler.

### Copy Number Inference From RNA-Seq Data

In order to identify the malignant cells in the cells drawn from patients with hepatocellular carcinoma, we compared the cancer cell chromosomal gene expression pattern with the putative non-cancer cells. The R package used here is infercnv (21) (version 1.2.1). First the human genome annotation file from the gencode database (https://www.gencodegenes.org/human/) was downloaded and converted to a genome position file. Then, the expression profiles of normal liver tissues provided by healthy donors were used as a reference, and the HCC group and the cirrhotic patient group were used as the observation group, since the data were 10x single-cell data, usually the cutoff is set as 0.1, denoise = T. Here, the copy number variation analysis was performed on 15 clusters according to the clustering model of Seurat.

### TCGA Data Analysis to Validate Results From Single Cell mRNA Sequencing

LIHC’s mRNA expression data and clinical data in TCGA were downloaded from UCSC Xena (http://xena.ucsc.edu/) database. We extracted the expression values of tumor associated genes from the TCGA mRNA expression matrix, combined with clinical data as input data. The univariate Cox regression analysis was used to screen tumor associated genes with OS values using the R package “survival” (version 3.1-8). The threshold of significance in all methods was set at value of p < 0.05. The multi-Cox regression analysis was used to establish prognostic models. In addition, Kaplan Meier (KM) survival curves were generated to graphically exhibit the prognostic outcomes between high and low risk groups that were divided through the median of the risk score. The proportion of 22 infiltrated immune cell types in patients was obtained by CIBERSORT (22). For correlation analysis, we used the proportion of the plasma cells and the proportion of total T cells. Spearman correlation between cell proportions were calculated with *cor* function in R. The relationship between the proportion of plasma cells with survival was also achieved using R package survival, and R package survminer was used to calculate the optimal threshold.

## Results

### Landscape of the Cell Composition and Characterization of Liver Tissue Cells in Healthy, Cirrhotic, and Cancerous Liver Tissues

**Figure 1:**
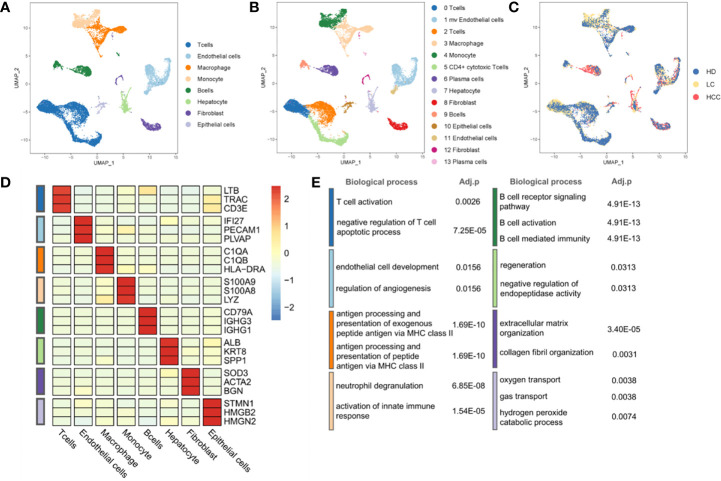
Landscape of liver tissue cells from healthy, cirrhotic and cancerous status. **(A)** The cells are divided into eight types, including T cells, Endothelial cells, Macrophages, Monocytes, B cells, Hepatocytes, Fibroblasts, and Epithelial cells. **(B)** Cell clustering through U-map dimensionality reduction, in which 14 clusters are divided, including three subtypes of T cells, two subtypes of Endothelial cells, two subtypes of Fibroblasts, two subtypes of B cells. **(C)** UMAP plot (in color) of 13,872 single cells from healthy donors (HD), liver cirrhosis (LC) and patients with hepatocellular carcinoma (HCC). **(D)** Heatmap of marker gene expression of eight cell types. **(E)** GO functional enrichment analysis of markers in eight cell types, p.adjust < 0.05. P.adjust were calculated using Benjamini and Hochberg method. (The color of the legend in **(D, E)** is consistent with that in **(A)**.

To describe the composition and function of cells of liver tissues at different status, we collected single cell transcriptome datasets from liver tissues of healthy donors, patients with cirrhosis and HCC. Through the analysis of single cell transcriptome data on the progression of HCC, we studied the composition of different cell types and cell states. We conducted normalization and then applied principal-component analysis (PCA) based on the highly variable genes (HVGs) (k = 2000) across all cells to implement dimensionality reduction by projecting the original transcriptomic profiles to the eigenvector space. With the linearly uncorrelated principal components (PCs) (k = 20), we performed Uniform Manifold Approximation and Projection (UMAP) analysis, which is a technique well-suited for visualization of high-dimensional data in a two-dimensional space. Cells from different sources overlap to form different clusters. The existing cell types in the original data set were taken as a reference, and the marker genes of various cells were used to annotate the cell groups. Cells were divided into eight categories and cell types were annotated (**Figure 1A**), according to the expression level of marker genes (**Figure 1D**, **Table S1**). There are four types of immune cells, T cells (represented by marker genes: LTB, CD3D, CD3E), Macrophages (C1QA, C1QB, HLA-DRA), Monocytes (S100A8, S100A9, LYZ), and B cells (CD79A, IGHG1, IGHG3). Four types are non-immune cells, including endothelial cells (IFI27, PECAM1, PLVAP), Hepatocytes (ALB, KRT8, SPP1), fibroblasts (SOD3, ATCA2, BGN), and epithelial cells (STMN1, HMGB2, HMGN2).

Through the GO functional enrichment analysis of eight groups (all p.adjust < 0.05), it was found that the T cell activation was enriched in T cells population, the endothelial cell development was significantly enriched in endothelial cells population. Macrophages, as typical antigen presenting cells, was enriched in the antigen processing and presentation of exogenous peptide antigen *via* MHC class II. In monocytes, the enrichment of genes was related to neutrophil aggregation and activation. Genes associated with B cell receptor signaling were found in B cell population. The hepatocyte population enriched in the regeneration pathway contains hepatocytes derived from cirrhosis. However, hepatocytes derived from cirrhosis in an inflammatory environment are characterized by regeneration and proliferation. This environment puts large numbers of hepatocytes at risk for the development of transforming mutations, and inexorably progresses to HCC (23, 24). This suggests that we may be able to find key clues to the transformation of cirrhosis into hepatocellular carcinoma in the hepatocyte population located in a cirrhotic TME. In addition, genes associated with the collagen fibril organization were found in fibroblasts population. The oxygen transport was enriched in epithelial cells population (**Figure 1E**, **Table S2**). The results of enrichment analysis provided strong evidences to support the cell annotation.

Further, eight cell populations were divided into 14 subclusters according to the Seurat clustering result, including three T cell subgroups, two B cell subgroups and other subgroups (**Figure 1B**, **Figure S1**). Many cancer-related fibroblasts, tumor-related endothelial cells and liver cells, but also a large number of B cells in HCC cells (**Figures 1A, C**) can be seen.

### The Role of Plasma Cells in the Tumor Microenvironment

**Figure 2:**
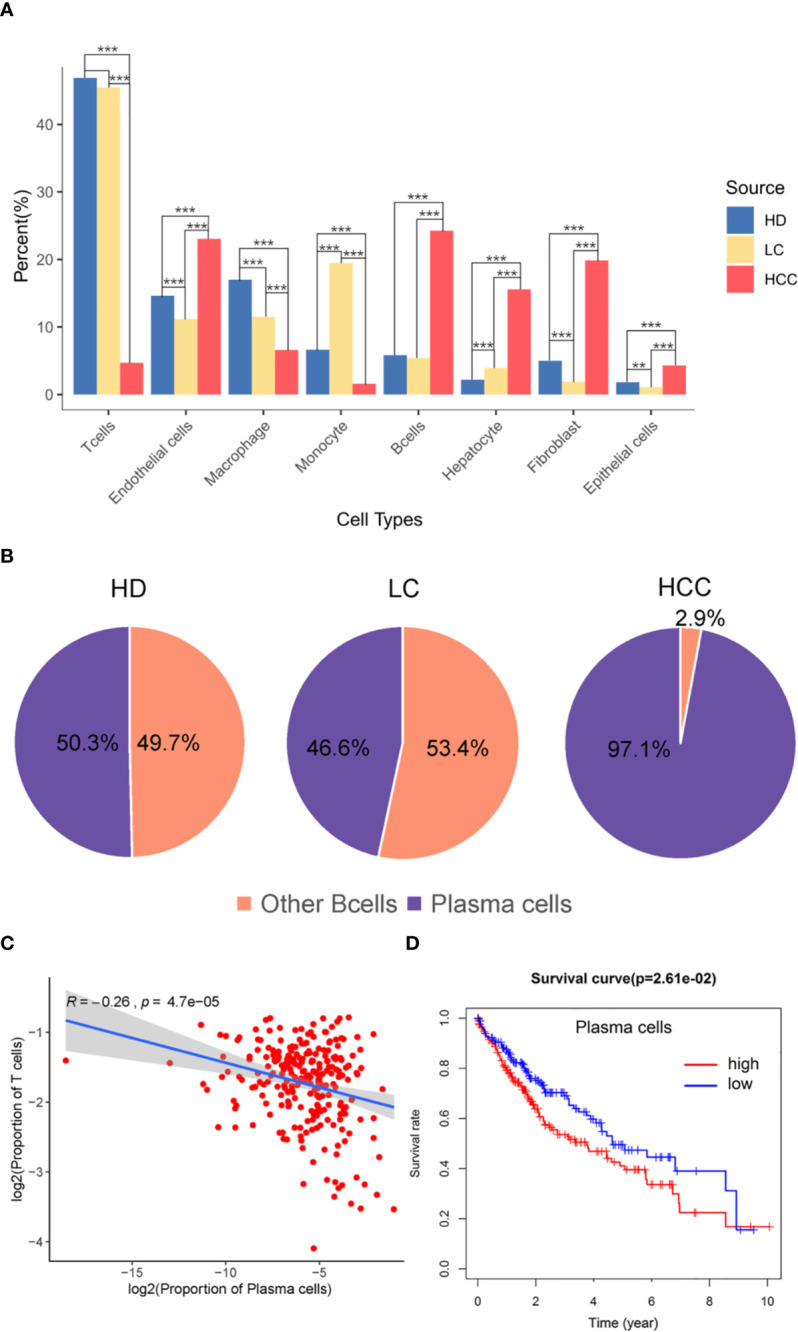
The role of plasma cells in the tumor microenvironment. **(A)** Bar plot showing the cell percent of different cell types in three sources, healthy donors (HD), patients with liver cirrhosis (LC), and patients with hepatocellular carcinoma (HCC). * p.adjust < 0.05, ** p.adjust < 0.01, and *** p.adjust < 0.001 (Fisher’s exact test). P.adjust values were calculated using Benjamini and Hochberg method. **(B)** The proportion of plasma cells and other B cells in the three sources. **(C)** Correlation of the plasma cells signature with T cells (TCGA LIHC data). Each dot represents a patient (Pearson’s correlation analysis). **(D)** Correlation between the proportion of plasma cell with survival rate, red is high proportion, blue is low proportion, p < 0.05 (Cutoff point = 0.008).

The proportion of different cell types were analyzed then, we hypothesized that the proportion distribution change might be associated with the disease status alteration. We studied the proportion of each cell type in the three sources, liver tissues of healthy donors (HD), patients with liver cirrhosis (LC), and patients with hepatocellular carcinoma (HCC). Compared with the cells in healthy donor, except for monocytes, the proportion of immune cells such as T cells, B cells, and macrophages decreased 1.40%, 5.49%, 0.41% in liver cirrhosis, respectively. While compared with the liver cirrhosis, the proportion of B cells in HCC increased 18.85% (p.adjust < 0.001, Fisher exact test, **Figure 2A**), T cells and other immune cells decreased (p.adjust < 0.001).

T cells and B cells have always played pivotal roles in tumor microenvironment. Here we observed the proportion of these two types of cells, and found the proportion of B cells from HCC (24.26%) liver tissue was significantly higher than that from healthy (5.82%) and cirrhotic (5.41%) liver tissue (p.adjust < 0.001). The proportion of T cells from HCC (4.71%) liver tissue was significantly lower than that from healthy (46.89%) and cirrhotic (45.49%) liver tissue (p.adjust < 0.001) (**Figure 2A**). B cell population in HCC are divided into two subgroups, B cells and plasma cells, and plasma cells accounted for 97.1% of the number of B cells (**Figure 2B**). Macrophages in HCC are tumor associated macrophages (TAMs), based on marker genes C1QA, C1QB (**Figure 1D**), co-express M1, and M2 signals (e.g. M1:CD64, M2: MACRO) (**Figure S2**) (2, 25). When M2 macrophages are present, plasma cells are tumor-promoting and have inhibitory effects on T cells (22, 26). The high proportion of plasma cells and the low proportion of T cells suggest that plasma cells may have a certain effect on the number of T cells. We further analyzed the infiltration proportion of immune cells in TCGA samples, such as plasma cells versus T cells, and the correlations between the infiltration proportion of plasma cells and total T cells was analyzed. It was found that the proportion of plasma cells has a negative regulatory relationship with the proportion of T cells. (R=-0.26, P=4.7e-05). In previous studies, it was also proved that in human HCC tissues, the number of plasma cells (>75% of them were IgG+) did inversely correlate with that of CD8+ T cells (27). To obtain a global understanding of the relationship between plasma cell population and the patient survival rate, a Cox proportional hazards model was applied, and the patients with lower proportion of plasma cells show a higher chance of survival (**Figure 2C**).

From the above results, it can be seen that plasma cells are related to the prognosis of patients with HCC, attention should be paid to plasma cells in the tumor microenvironment of HCC. Plasma cells should be considered to be potentially related to the occurrence and progression of HCC.

### Construction of the Differential Trajectories of B Cells

In order to further explore the status of B cells in healthy, cirrhotic, and cancerous liver tissue microenvironment, we simulated the trajectory of B cells from three sources and observed the differentiation of B cell population.

**Figure 3:**
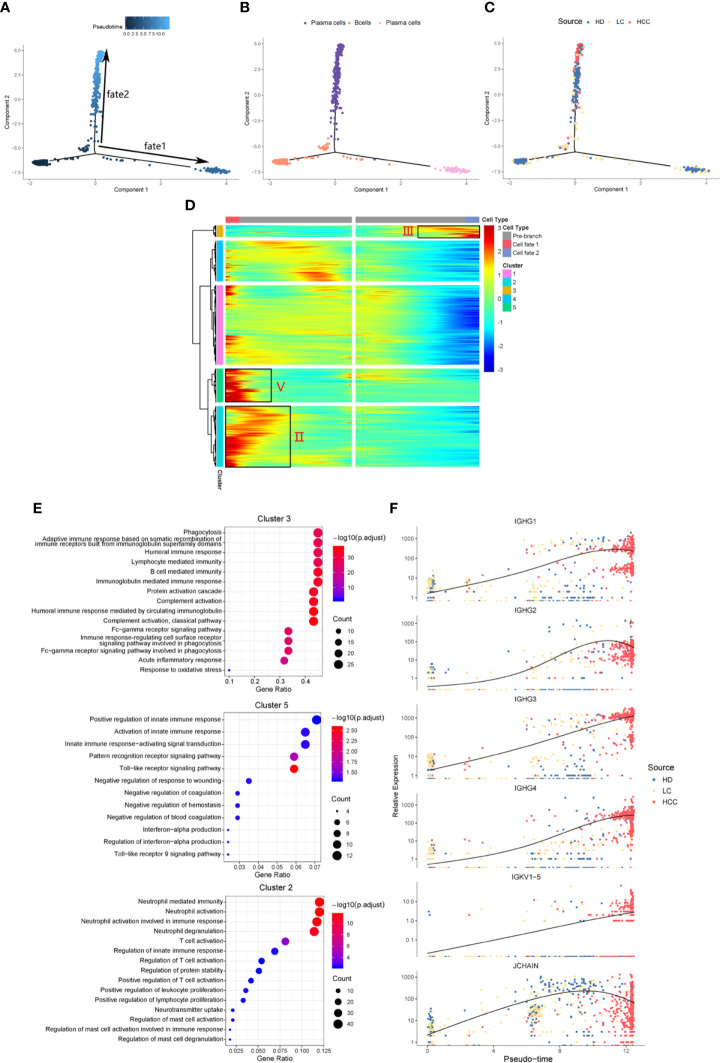
Simulation of the development trajectory of B cells and the analysis of gene expression pattern. Simulation of the differentiation trajectory of B cells group from three sources, healthy donors (HD), patients with liver cirrhosis (LC), and patients with hepatocellular carcinoma (HCC), **(A)** pseudo-trajectory of B cells, **(B)** cell type transition, **(C)** cell source transition. **(D)** Heatmap shows the gene expression dynamics of B cell group. Genes (rows) are clustered and cells (columns) are ordered according to the pseudotime development. Genes are listed in **Table S2**. Gene clusters III, V, and II were selected for further analysis. **(E)** The GO function enrichment analysis results of gene cluster III, V and II of B cell group, p.adjust < 0.01. (The first 12 GO terms are retained). **(F)** Expression patterns of some genes enriched in humoral immunity under three sources.

We identified the differentially expressed genes in B cell population between healthy donors and patients with liver cirrhosis and liver cancer. R package monocle was then used to sort individual cells by these genes and construct the tree-like structure of the entire lineage differentiation trajectory (**Figure 3A**). From the perspective of cell typing, the starting point of branching is composed of B cells, and the two other branches are composed of plasma cells (**Figure 3B**), this trend is consistent with the differentiation process from B cells to plasma cells. From the perspective of cell sourcing, the starting point for cell differentiation (pre-branch) consists of a large number of cells from healthy donors, patients with liver cirrhosis, only very few cells from patients with HCC. The cell composition of cell fate 1 is similar to that of pre-branch. The cells at the beginning and middle of fate branch 2 were mainly from healthy donors and patients with cirrhosis, and almost all the cells at the end are from patients with HCC (**Figure 3C**). We performed branched heatmap to show the gene pattern of different cell fate branches, based on the expression dynamics, genes are divided into five clusters according to their expression patterns (**Figure 3D**). The gene cluster V (cluster 5) and II (cluster 2) were highly expressed in cell fate1, III (cluster 3) were highly expressed in cell fate 2. From the results of GO enrichment in B cell group, it was found that humoral immunity and B cell mediated immunity-related GO terms are enriched in gene cluster III (**Figure 3E**), regulation of innate immune response and neutrophil activation GO-related terms are enriched in gene cluster V (cluster 5) and II (cluster 2) (**Figure 3E**).

We also analyzed the expression patterns of some genes enriched in humoral immune GO term (**Figure 3F**). Representatively, B cell and humoral immune response marker gene IGHG1 demonstrates a gene expression pattern of gradual up-regulation from normal cells to cancer cells, and then slightly droops. Similar patterns can be observed in IGHG2, IGHG4, and JCHAIN. Often patients with chronic liver disease have higher humoral immune index than healthy people (28, 29). This suggests that attention should be paid when the humoral immune index of patients with liver cirrhosis slows down after a rapid increase, it may be a dangerous signal for cirrhosis developing into HCC. This result might indicate that humoral immunity displays compensatory increase during tumorigenesis, and could be used as an important index for early HCC surveillance.

### Tumor Prognosis Associated Genes Identified in Single-Cell Gene Expression Patterns of Hepatocytes

We conducted an analysis of copy number variation (CNV) for hepatocytes from each group of tissue source. Based on gene expression profiles aligned along the chromosomes as moving averages, we used chromosomal gene expression patterns to identify the malignant cells of hepatocytes (30, 31). With this approach of recapitulating tumor-specific CNVs (31), malignant cells would show distinct chromosomal expression patterns.

**Figure 4:**
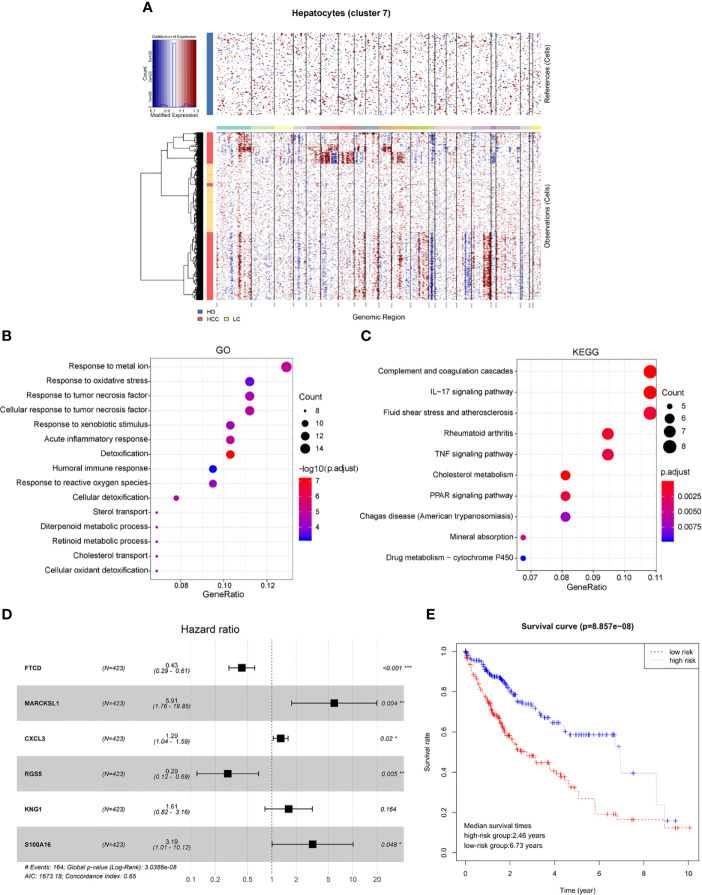
Identification of HCC prognosis associated genes from malignant hepatocyte single cell cluster. **(A)** Copy number variation in hepatocyte group, the healthy group (healthy donor, HD) was used as a reference, the cirrhosis group (Liver cirrhotic patient, LC), and the HCC group (HCC patient) were used as observation groups, red represents overexpression of genes and blue represents low expression. **(B)** The result of GO functional enrichment analysis, p.adjust < 0.01. **(C)** The result of KEGG pathways enrichment analysis, p.adjust < 0.01. **(D)** Hazard ratio of the six genes. **(E)** Kaplan-Meier estimates of overall survival from HCC patients in TCGA based on six-gene signatures, patients were divided into low-risk group and high-risk group according to median risk score.

Genomic expression in liver tissue cells from healthy liver donors (HD), patients with Liver cirrhosis (LC), and patients with hepatocellular carcinoma (HCC) were compared on the basis of chromosomal gene expression patterns, the HD group of cells was used as a reference, the HCC group and the liver cirrhosis group were used as the observation group. We found that the copy number of cells provided by patients with HCC in hepatocytes (cluster 7) had a significant change (**Figure 4A**), so we believe that this hepatocyte cluster 7 were malignant HCC cells.

Therefore, through differential expression analysis, we identified a total of 119 marker genes in HCC cells from hepatocyte cluster 7. The GO functions were enriched in terms such as acute inflammatory response, humoral immune response and response to oxidative stress (**Figure 4B**), while the KEGG pathways were enriched to such as IL-17 signaling pathway and TNF signaling pathway (**Figure 4C**). The above results may indicate a compensatory phenomenon, suggesting that these genes may be associated with the tumorigenesis and progression of tumors.

Next, the bulk data in TCGA was used to identify HCC related prognostic markers out of this group of genes. The mRNA expression data of HCC in TCGA and the clinical data of 438 patients of LIHC were downloaded. The 119 differentially expressed genes were extracted from the mRNA expression data of TCGA for Univariate Cox hazard analysis. Single gene risk ratio and p-value were obtained, of which 11 genes (FTCD, ADH4, MARCKSL1, SPARCL1, CXCL3, HULC, RGS5, SORBS2, HPX, KNG1, S100A16) were identified significantly related to the patients’ overall survival (p < 0.01). Next, six genes, FTCD, MARCKSL1, CXCL3, RGS5, KNG1, and S100A16, were screened out by the Akaike information criterion (AIC) to construct a prognostic model. It is worth noting that FTCD is associated with autoimmune hepatitis, and S100A16 plays an important role in the development of various malignant tumors. Then the Multivariate Cox hazard analysis model was constructed, and Hazard ratio of each gene is shown (**Figure 4D**, Hazard Ratio (HR)> 1 is considered high risk, HR <1 is low risk). The model can predict the risk value of each patient, and draw a risk curve according to the patient’s high and low risk values (**Figure 4E**). Taking the median of the risk score as the threshold value, the samples are divided into two groups: high-risk group (n = 203) and low-risk group (n = 207). The median survival times are 2.46 years in the high-risk group vs. 6.73 years in the low-risk group, p < 0.001. The results show that the synergy of these six genes identified from single malignant hepatocytes plays an important role in the progression of HCC associated with cirrhosis.

## Discussion

Single-cell RNA-seq (scRNA-seq) provides a cutting-edge method to study the cellular heterogeneity of the tumor microenvironment of many cancer types, by profiling the transcriptomics of thousands of individual cells. Bulk-tissue-level resolution may mask the complexity of alterations across cells and within cell groups, especially for less abundant cell types (32). ScRNA-seq analysis can help better understand cellular collective behavior and mutual regulatory mechanisms within a tissue ecosystem. Currently, studies on the relationship between cirrhosis and HCC at the single-cell level are still unclear. In our study, transcriptional level sequencing data of more than 20,000 single cells were collected and various cell types were analyzed, providing a new perspective for understanding the cell composition characteristics and pathogenesis development in the microenvironment of liver cirrhosis and HCC.

The microenvironment of cirrhotic and cancerized liver tissues are characterized with extensive immune infiltration. T cells and B cells are the most abundant and best-characterized population in tumor microenvironment (TME) of solid tumors (6). T cells are always the focus of anti-tumor activity research of HCC. A recent study found 11 T cell subsets of HCC based on their molecular characteristics through large-scale single-cell transcriptome sequencing (33). Meanwhile, the optimal efficacy of chimeric antigen receptor T cells in the immunotherapy of solid tumors inspired the research of CD8+T cells in HCC (34). The pivotal role of T cells in anti-tumor responses has been widely studied and well established. However, the importance of the immune response of B cells to tumorigenesis and development is not clear yet. B cells represent a heterogeneous population with functionally distinct subsets, contributing to both pro-tumor as well as anti-tumor immune responses, and the balance among the subtypes may affect tumor development and behavior (35, 36). Previous study demonstrated that B cells in tumor microenvironment are related to immune responses. Recent evidences show that B cells have the capacity to recognize antigens, regulate antigen processing and presentation, and mount and modulate T-cell and innate immune responses (37). In our study, we found that there may be a negative regulatory relationship between the number of plasma cells and T cells in the TME. It’s been reported recently, B cells may generate inhibitory factors that dampen the response of other immune cells, or generate molecules on the surface of B cells that hinder the targeting and destruction of tumor cells. The above process may cause tertiary lymphoid structures (TLS) in tumors to be immature TLS. If B cells have less interaction with T cells and greater interaction with malignant tumors, these two inhibitory mechanisms may appear at the same time. There are now three studies (38–40) that provide indirect evidence that immature TLS is associated with low T cell activity in tumors (41). In our study, plasma cells from HCC accounted for 97.1% of B cells, it was also found from the analysis of clinical data that the patients with lower proportion of plasma cells had higher chance of survival. Our results therefore provided a supportive reference for the impact of B cells on the occurrence and development of solid tumors.

From the clinical perspective, most patients with liver cancer have an anamnesis of hepatitis or cirrhosis, cirrhosis is a major risk factor for the development of HCC. Early detection of HCC associated with cirrhosis is a challenge and remains critical in guiding the optimal clinical treatment of the disease. Therefore, the presence of suggestive markers and diagnostic biomarkers for early events during the development of HCC is very valuable for early detection. The humoral immune index of patients with cirrhosis and other chronic liver diseases are higher than those of healthy liver. Our research found that changes in humoral immune index may be an important hint for the conversion of cirrhosis into HCC. By simulating the changes in cell sources and cell types in the cell differentiation trajectory, we found that a large number of plasma cells aggregated under the source of HCC. Functionally, plasma cells show potency to produce antibodies to participate in the humoral immune process. When the changes of enrichment genes in humoral immunity were investigated, it was also found that these genes were continuously upregulated in liver status from the healthy to cirrhosis to carcinoma (HCC), and there was a peak value in the liver status of HCC and then droops. Therefore, we believe that when the rise of humoral immunity index slows down after reaching a peak, it should be noted that there may be a risk of cancerization.

Through inferring the copy number variation of single-cell transcriptome data, the malignant and non-malignant cells of hepatocytes were separated. The tumor associated genes between malignant hepatocytes and cirrhotic hepatocytes were identified. We find that these genes were enriched in the GO terms, including humoral immune response and response to oxidative stress, etc., same with the highly expressed gene cluster III in B-cell differential trajectory fate2. JUN, a transcription factor, was differentially expressed both in tumor associated genes in hepatocytes and highly expressed gene cluster 3 in B-cell differential trajectory fate2. It is strongly expressed with inflammatory stimulation, promotes hepatocyte survival during acute hepatitis and acts as an oncogene in the process of chemically inducing mouse cancer. JUN plays an important role in transformation of liver from inflammation to tumor (42). All these results suggest that there may be a relationship between hepatocytes and B cells in HCC development, and further studies on their interaction can be carried out in the future. In addition, JUN is also a positive regulator of T cells, which can affect the changes of T cell receptor signaling pathway (43). These tumor associated genes combined with bulk RNA-seq data helped us screen out prognosis related molecular markers for HCC. In the six-gene prognostic modeling, four genes are the major contributors with HR>1, MARCKSL1, CXCL3, KNG1, S100A16 (p < 0.05). Studies have shown that these genes play roles in promoting various cancers. MARCKS/MARCKSL1 overexpression could restore the self-renewal of liver tumor–initiating cells (TICs), TICs form small subsets of cells in hepatocellular tumors and account for tumorigenesis, metastasis, recurrence, and drug resistance (44). S100A16 was expressed in lung adenocarcinoma (AC) tissues with poor prognosis. CXCL3 can be a potential target for precise therapy of HCC (45). KNG1 was demonstrated as a biomarker in the occurrence of HCC (46). The above reports combined with the results of this study suggest these genes could be potential cellular candidates for therapeutic targeting in multiple types of cancers.

In summary, our comprehensive characterization of cells at single level from different status of liver tissues revealed cell composition nature and gene expression pattern in both liver tissue microenvironment and liver malignant cells. Changes in humoral immunity, including plasma cells proportion increasing, and marker gene expression pattern of climbing and drooping, may be indicative surveillance for the alteration from cirrhosis to poor prognostic HCC. The potential prognostic biomarker signature constructed with tumor associated genes identified in hepatocytes comparison among HD, LC and HCC, might be conducive to developing therapeutic strategies.

## Data Availability Statement

The datasets presented in this study can be found in online repositories. The names of the repository/repositories and accession number(s) can be found in the article/**Supplementary Material**.

## Author Contributions

LX conceived of the idea, and planned and coordinated the entire project. LX and LC supervised this study. ZL contributed to the study design. SZ conducted data analysis and figure generation. ZL provided part of the data analysis code. SZ drafted the manuscript. LX, ZL, and DW revised the manuscript. All authors contributed to the article and approved the submitted version.

## Funding

This work was funded by Shanghai Municipal Health Commission, and Collaborative Innovation Cluster Project (No. 2019CXJQ02) and National Natural Science Foundation of China (No. 31870829).

## Conflict of Interest

The authors declare that the research was conducted in the absence of any commercial or financial relationships that could be construed as a potential conflict of interest.

## Supplementary Material

Supplementary Figure 1 Heatmap of expression of highly expressed genes in the results of Seurat clustering (14 clusters).

Supplementary Figure 2 Gene marker expression plot for classical macrophage cell types M1 (CD64), M2 (MARCO).

Supplementary Figure 3 The expression of six tumor associated genes in hepatocyte cluster in three sources, healthy donors (HD), patients with liver cirrhosis (LC), and patients with hepatocellular carcinoma (HCC).

Supplementary Table 1 Gene markers of eight cell types.

Supplementary Table 2 GO functional enrichment results corresponding to 8 cell types (p.adjust < 0.05).

Supplementary Table 3 Five groups of genes with different expression patterns in the differentiation trajectory of B cell population.
