# Supplementary figures and images for: Single-Cell RNA-Seq Analysis Reveals Microenvironmental Infiltration of Plasma Cells and Hepatocytic Prognostic Markers in HCC With Cirrhosis
Source: Front Oncol. 2020 Nov 2;10:596318. doi: 10.3389/fonc.2020.596318 (PMC7667372; doi:10.3389/fonc.2020.596318)

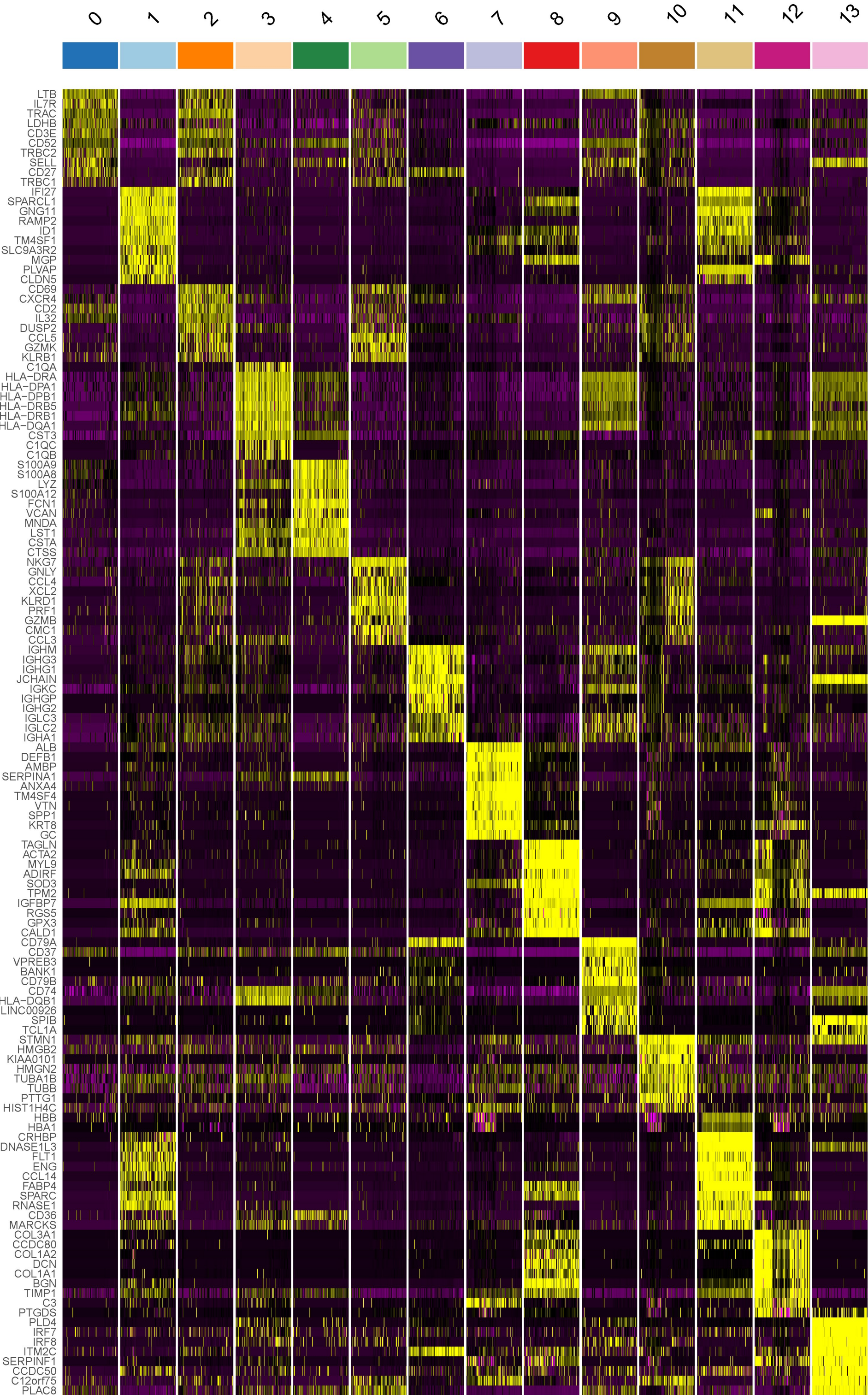

Supplement: Supplementary Figure 1 — Heatmap of expression of highly expressed genes in the results of Seurat clustering (14 clusters). [file Image_1.pdf]

**CD64(FCGR1A)**

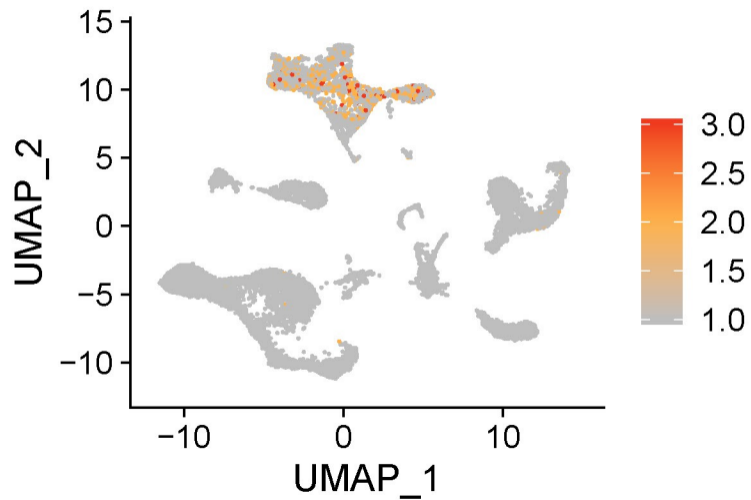

**rna\_MARCO**

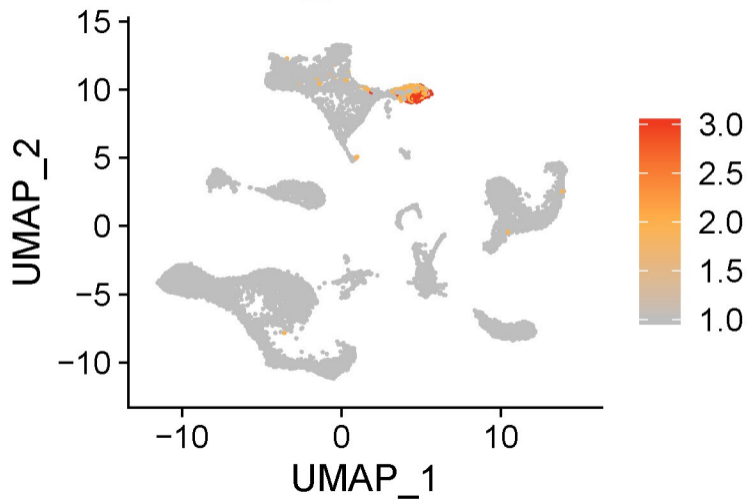

Supplement: Supplementary Figure 2 — Gene marker expression plot for classical macrophage cell types M1 (CD64), M2 (MARCO). [file Image_2.pdf]

FTCD

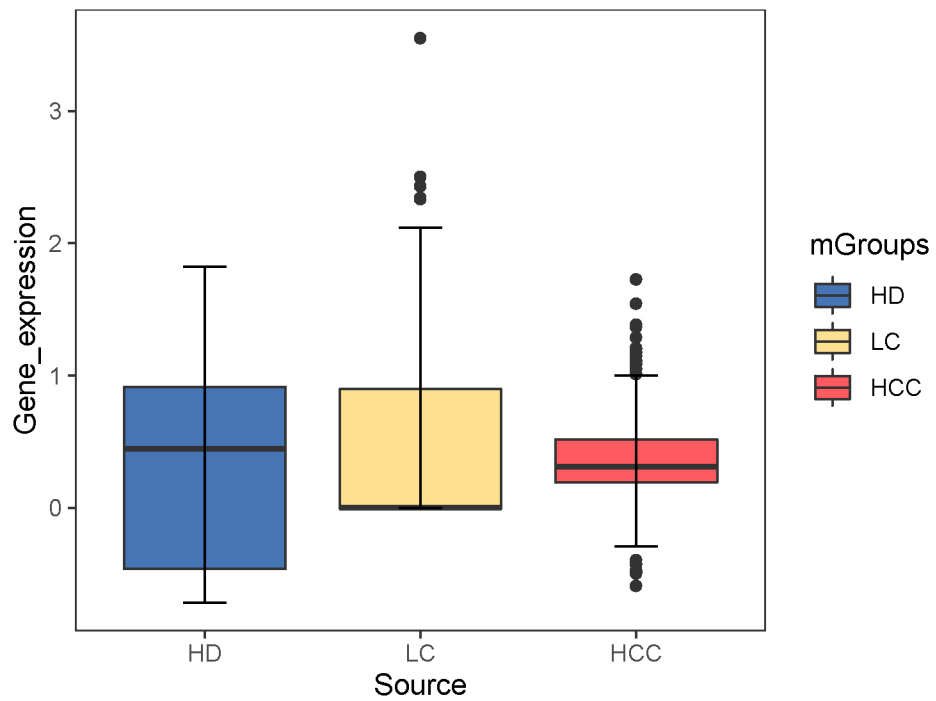

MARCKSL1

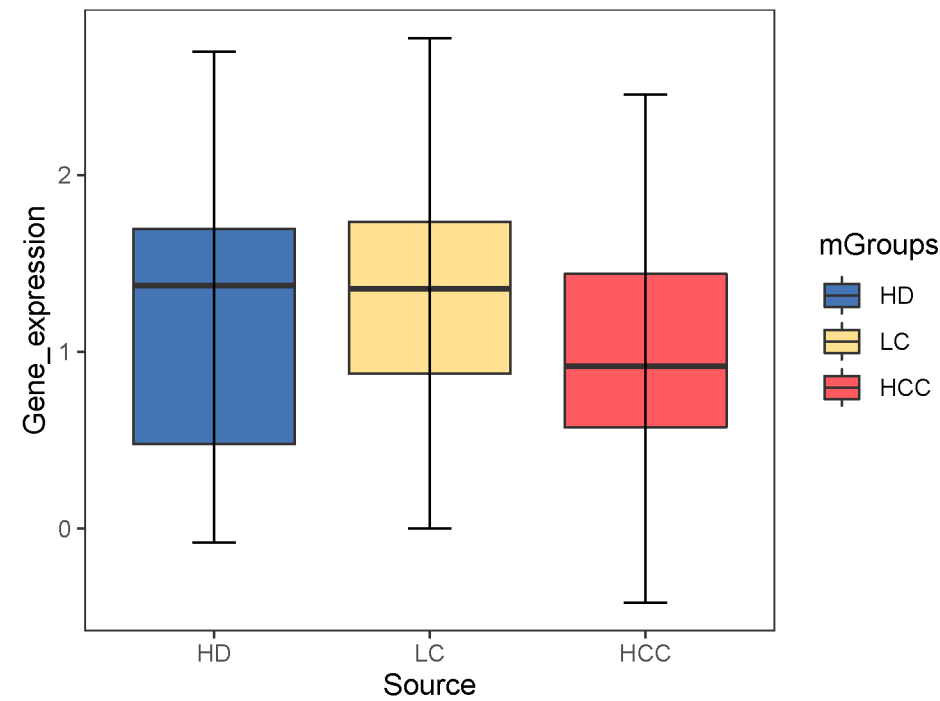

CXCL3

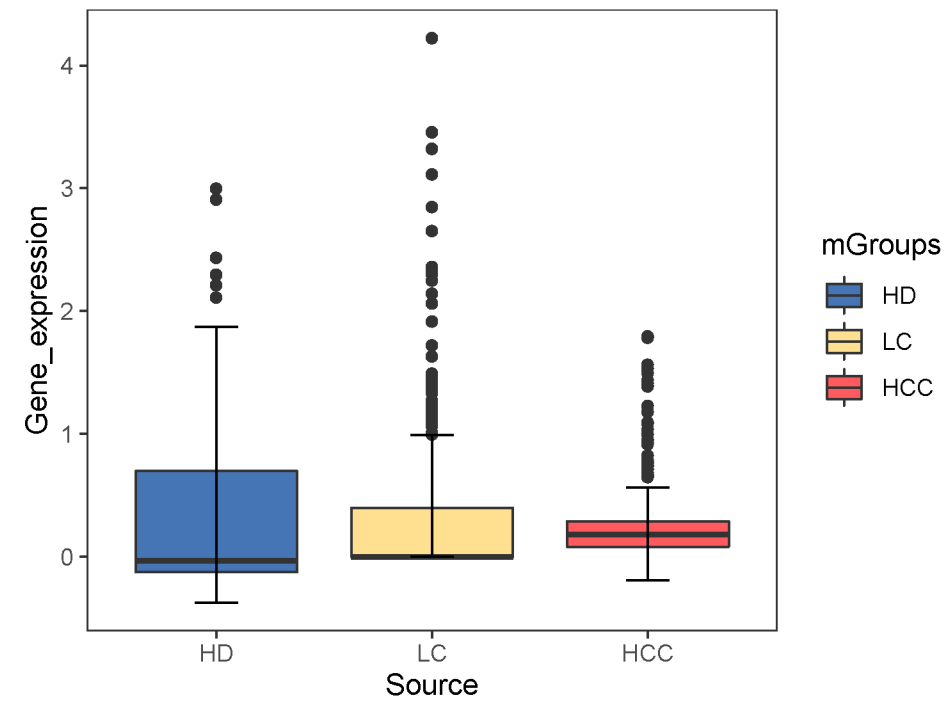

RGS5

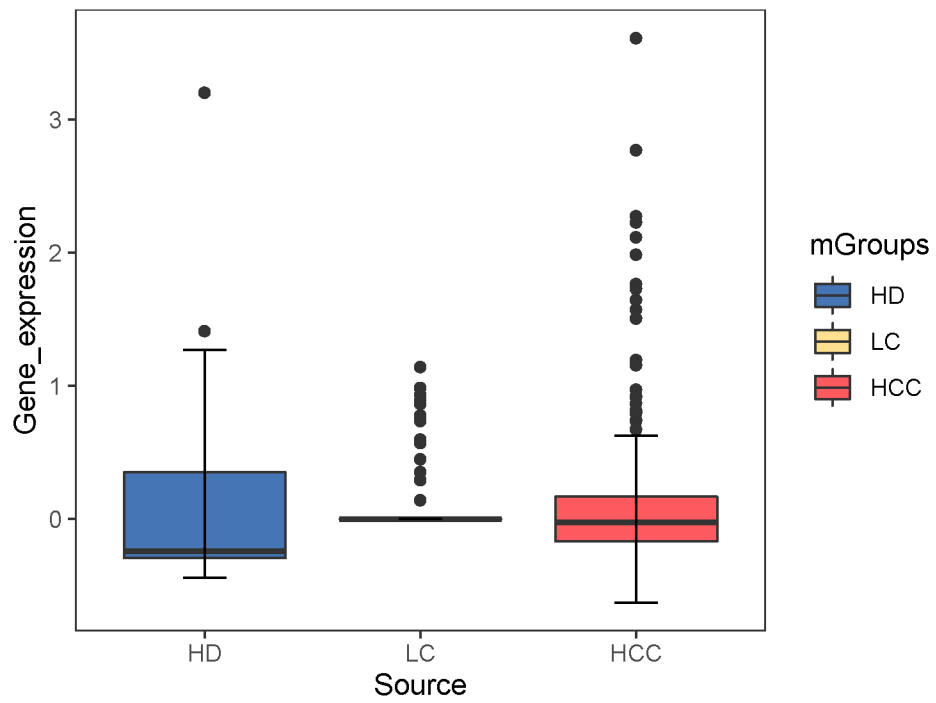

KNG1

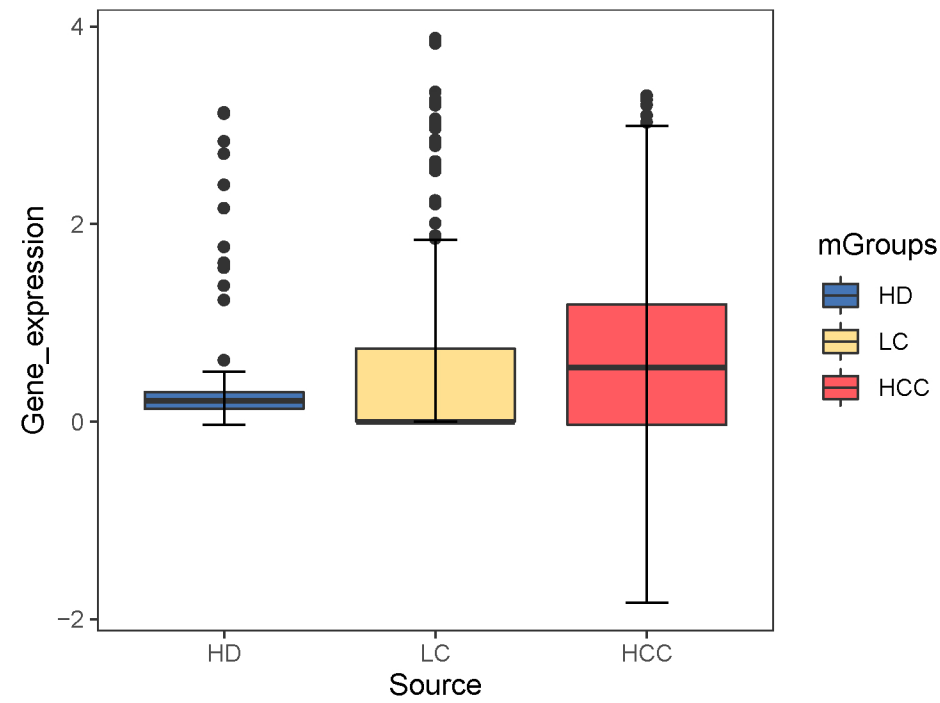

S100A16

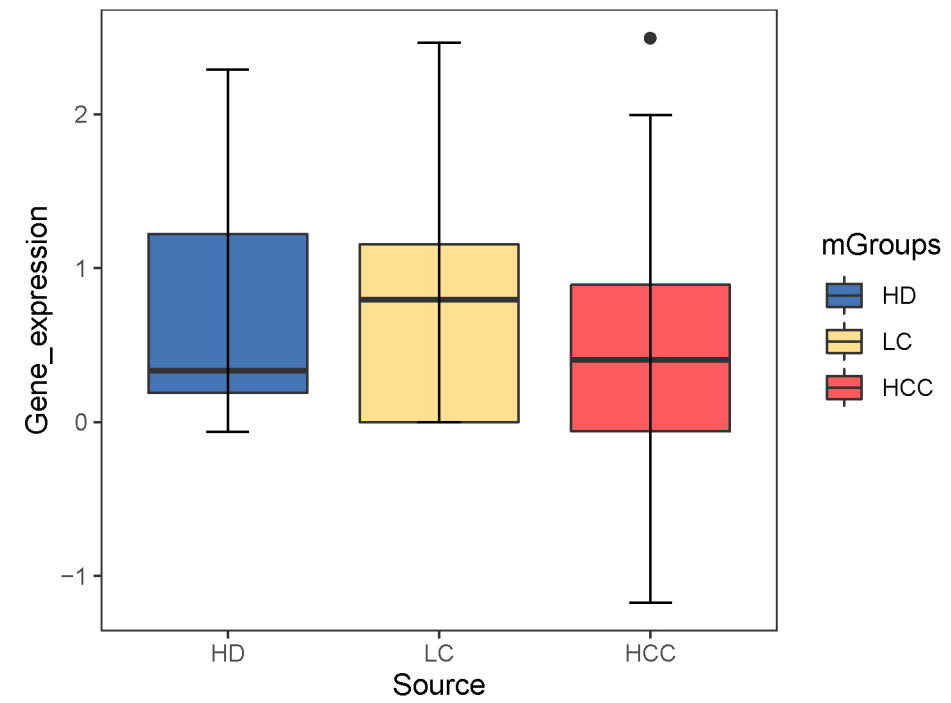

Supplement: Supplementary Figure 3 — The expression of six tumor associated genes in hepatocyte cluster in three sources, healthy donors (HD), patients with liver cirrhosis (LC), and patients with hepatocellular carcinoma (HCC). [file Image_3.pdf]
